# Antimicrobial resistance in a protracted war setting: a review of the literature from Palestine

**DOI:** 10.1128/msystems.01679-24

**Published:** 2025-05-21

**Authors:** Ramya Kumar, Osama Tanous, David Mills, Bram Wispelwey, Yara Asi, Weeam Hammoudeh, Omar Dewachi

**Affiliations:** 1 University of Chicago Pritzker School of Medicine, Chicago, Illinois, USA; 2 FXB Center for Health and Human Rights, Harvard University, Boston, Massachusetts, USA; 3 Department of Pediatrics, University of California San Diego School of Medicine, La Jolla, California, USA; 4 Harvard Medical School, Harvard University, Boston, Massachusetts, USA; 5 School of Global Health Management and Informatics, University of Central Florida, Orlando, Florida, USA; 6 Institute of Community and Public Health, Birzeit University, Birzeit, Palestine; 7 Department of Anthropology, Rutgers University, New Brunswick, New Jersey, USA; The University of Maine, Orono, Maine, USA

**Keywords:** AMR, war, Palestine, equity, settler colonialism

## Abstract

Antimicrobial resistance (AMR) is an escalating global health crisis. In war-affected regions, where healthcare systems are already compromised, AMR presents an even greater threat. The occupied Palestinian territories (oPt), subjected to chronic war-like conditions, have experienced extensive fragmentation, de-development, and destruction of healthcare. This review maps the existing knowledge regarding AMR carriage and infection in the oPt. We conducted a scoping review using PubMed, CINAHL, Embase, and Web of Science. Findings on the prevalence and suggested drivers of AMR were extracted and analyzed according to the One Health approach. From an initial 1,787 articles, 102 were included in the final analysis. Of these, 91 studies focused on AMR in humans, seven on animals, and four on water contamination. The findings reveal high rates of AMR, with 40% of *Klebsiella pneumoniae* producing extended-spectrum beta-lactamase (ESBL) and 55% of *Streptococcus pneumoniae* resistant to penicillin. Additionally, 45.6% of *Staphylococcus aureus* isolates from cow milk were methicillin-resistant, and 36% of chicken samples contained carbapenem-resistant gram negative bacteria. Significant gaps in the regulation of antibiotic prescription, dispensation, and consumption were reported. Despite the heterogeneity in reporting methods across studies and the inability to perform a meta-analysis, the evidence suggests alarmingly high AMR rates within the oPt. Notably, most studies attributed AMR to local practices, often overlooking broader structural and socio-political drivers. This review underscores the need to contextualize AMR within the environment, particularly under conditions of sustained warfare.

**IMPORTANCE:**

This study goes beyond merely reviewing and summarizing data on AMR in the occupied Palestinian territories (oPt), a region enduring chronic war-like conditions. Our work addresses critical gaps in knowledge about AMR in populations affected by war and siege. By contextualizing AMR through a socio-political lens, this study offers a novel perspective. It highlights deeper drivers, including the impact of war on the behaviors and education of patients and doctors, perceptions of antibiotics, the role of humanitarian interventions in fostering antibiotic anarchy, and the overall weakening of the Palestinian healthcare system. Importantly, this review also sets the stage for understanding the existing literature on AMR in the oPt within the context of the ongoing war in Gaza, emphasizing the immediate need for comprehensive strategies to address AMR under conditions of conflict. The insights can inform physicians and policymakers in designing and implementing effective AMR stewardship programs, not only in Palestine but also in other conflict-affected regions.

## INTRODUCTION

Antimicrobial resistance (AMR) is a critical and growing global health crisis, affecting both healthcare and ecological systems. By 2050, AMR could potentially result in 10 million deaths and impose an economic burden of $100 trillion (1, 2). Countries affected by war and conflict, primarily in the Global South—such as the Democratic Republic of Congo (3), Afghanistan (4), Yemen (5), Syria (6), Iraq (7), and Ukraine (8) exhibit significantly higher rates of AMR compared with those unaffected by conflict (9, 10). This pattern suggests that war is a significant driver of AMR (11). In conflict-affected regions, AMR poses one of the greatest challenges to health and rehabilitation, leading to increased healthcare costs and poorer health outcomes (9, 12).

War and armed conflict exacerbate AMR through multiple pathways. Compromised healthcare infrastructure, inadequate AMR surveillance, and limited diagnostic and treatment options are major issues. The destruction of healthcare facilities further hampers the management of infectious diseases (13–15). Traumatic injuries, common in war zones, often result in complex wounds infected with AMR bacteria, prolonging hospital stays and heightening the risk of morbidity and mortality (13, 16, 17). Humanitarian aid, although necessary, sometimes contributes to “antibiotic anarchy” due to the uncontrolled distribution of antibiotics without proper oversight (18, 19). Additionally, heavy metal contamination from munitions and waste in conflict areas may drive bacterial mutations, further contributing to AMR (20).

The One Health approach emphasizes the interconnectedness of human, animal, and environmental health and advocates for multidisciplinary collaboration (21) to combat AMR (22). In war-affected regions, livestock exposed to contaminated water and feed can develop AMR, posing risks to human health throughout the food chain (22).

AMR driven by war is not confined to conflict regions. As refugees and asylum seekers flee to safer countries, they can carry the risk of spreading AMR globally. Additionally, soldiers returning from overseas wars may introduce AMR to their home countries (23).

### Setting: the Israeli occupied Palestinian territories (oPt)

Unlike the conventional war contexts mentioned earlier, Palestinians experience war as part of a broader and long-term project of settler colonization. This colonization is characterized by various forms of violence and control, including mass killings (24–26), forced expulsions and displacements (27, 28), apartheid, and systems of segregation that confine people to camps or restricted territories. These ongoing conditions have resulted in statelessness and a fragmented political status for Palestinians (29–31). Furthermore, the continued occupation has severely impacted living environments, water resources, and agricultural areas in the oPt (32–34). For Palestinians, war is not seen as a conventional conflict with a clear beginning and end; instead, it represents an enduring condition that shapes daily life and maintains settler colonization and military occupation with no end in sight (35, 36).

The Palestinian territories occupied by Israel in 1967 include the West Bank, East Jerusalem, and the Gaza Strip. Although these areas are categorized similarly under international law, they experience various forms of Israeli military occupation (37). This has resulted in distinct social, political, and ecological conditions, as well as fragmented and disconnected healthcare and therapeutic geographies across these regions (38–40).

The West Bank, situated west of the Jordan River, is fragmented by a network of checkpoints, Israeli settlements, and a separation wall. In the West Bank, the chronic war-like condition manifests as military occupation, frequent raids, home demolitions, land confiscation, settlement expansion, and strict control over the movement of people, as well as the import and export of goods (40). Additionally, the West Bank’s geographical connection with Jordan significantly influences the movement of medical supplies and the travel of patients seeking healthcare outside the West Bank (41).

The Gaza Strip, demarcated by the 1948 armistice line, has a population where 80% are refugees from historical Palestine. Similar to the West Bank, Gaza has been under Israeli occupation since 1967. Following Hamas’s electoral victory in 2006, Israel subjected the Gaza Strip to one of the longest and most severe sieges in modern history, severely restricting the movement of people, goods, food, electricity, fuel, and medical supplies (42, 43). The blockade has particularly impacted medical supplies and patient access to healthcare, with links to Egypt serving as a critical, yet heavily controlled, pathway for the movement of medical resources and patients seeking treatment outside of Gaza. This prolonged siege created a state of perpetual “biosphere of war” (44), marked by recurrent military operations, extensive bombing campaigns, significant casualties, and the destruction of infrastructure, including healthcare facilities (42, 45, 46).

The decimation of healthcare infrastructure, scarcity of medical supplies, and overcrowded conditions have severely impaired the ability to manage and prevent infections effectively. These conditions have been associated with a rise in AMR infections; during the 2018 Great March of Return (47), medical professionals reported that nearly all 2,000 patients admitted for gunshot wounds had AMR infections (48). These infections, linked to antibiotic-resistant bacteria, contributed to longer hospital stays and increased risk of amputations (13).

Before 7 October 2023, the situation in the Gaza Strip was already dire; afterwards it became catasrophic as Israel launched its most devastating war on Gaza, which has been deemed a genocide by a growing consensus of scholars and human rights groups and a “plausible genocide” in the ongoing ICJ case against Israel (49–51). By the time of writing, Israel has killed more than 50,000 Palestinians (52, 53), including more than 500 healthcare workers (54), and the extensive destruction of medical infrastructure (55, 56). Since then, conditions have drastically worsened, with most healthcare facilities destroyed, critical infrastructure demolished, and widespread outbreaks of infections and diseases. The population is now facing severe starvation and a humanitarian crisis of unprecedented scale (56, 57).

Despite their different conditions, both the West Bank and the Gaza Strip experience a fragmented healthcare environment marked by inadequate infrastructure and limited access to medical resources. Healthcare in these regions is provided by a variety of actors, including community-led healthcare committees, non-governmental organizations (NGOs), the United Nations Relief and Works Agency (UNRWA), private healthcare providers, humanitarian organizations, and the Palestinian Ministry of Health (MoH), which was established following the Oslo Accords in the early 1990s (39).

This diversity of healthcare providers, combined with a lack of regulation and the absence of a cohesive national policy on antibiotic use, fosters unregulated antibiotic consumption (39–41, 43, 58, 59). Additionally, ongoing attacks on healthcare facilities, such as the bombing of hospitals and laboratories, further disrupt the detection, treatment, and monitoring of AMR. Palestinians in both the West Bank and Gaza Strip face constant fear, insecurity, and uncertainty regarding healthcare and medication availability, which often leads to the overuse and hoarding of antibiotics (60).

Recognizing these challenges, a national action plan on AMR published by the World Health Organization (WHO) and the Palestinian Ministry of Health in 2020 highlighted that “AMR in Palestine acquires an additional dimension due to the prevailing conflict,” which leads to poor infection control, unregulated prescription practices, and easy access to antibiotics. The plan identified political instability and conflict as significant barriers to the successful implementation of a comprehensive national AMR strategy, emphasizing the growing urgency to address AMR in Palestine (61).

### Objectives

This scoping review aims to (i) identify and synthesize data on AMR carriage and infection among Palestinians living in the oPt, (ii) map existing knowledge on AMR in the oPt, and (iii) identify gaps in the current AMR literature in the oPt.

## METHODS

Initial literature searches were conducted in PubMed to identify relevant studies and refine search terms. Keywords and subject headings were extracted from these results through text mining. Search strategies were developed in PubMed using Boolean logic combined with mined keywords and subject headings, focusing on the concepts of Palestine and antimicrobial drug resistance. A sample search strategy is provided in the Supplemental Material, and additional search details are available upon request.

Further keywords were identified through text mining in Embase and other databases. The final search strategies were tested against known articles to ensure efficacy and were then adapted for use in the following databases: Cochrane Database, Cumulative Index to Nursing and Allied Health Literature (CINAHL), Embase, and Web of Science Core Collection. Additional gray literature searches were performed using Google Scholar.

Duplicate records were removed using the validated EndNote deduplication method by Bramer et al. (62). The results were imported into Covidence systematic review software, where each title and abstract were independently screened by two reviewers. Discrepancies were resolved through discussion. Full-text screening of the remaining studies followed the same method. Data extraction was performed using Microsoft Excel.

The inclusion criteria encompassed studies on AMR, including infection or colonization, conducted in humans, animals, and the environment within the occupied Palestinian territories (West Bank, East Jerusalem, and Gaza Strip). Studies involving Palestinian citizens of Israel or Palestinian refugees outside the West Bank and Gaza Strip were excluded, as were studies conducted outside the occupied territories or on non-Palestinian populations. Additionally, studies focusing on specific patient populations with frequent hospitalizations and high antibiotic use, such as those with malignant diseases, dialysis patients, and individuals with cystic fibrosis were excluded. Case studies of individual patients were also excluded.

## FINDINGS

**Fig 1:**
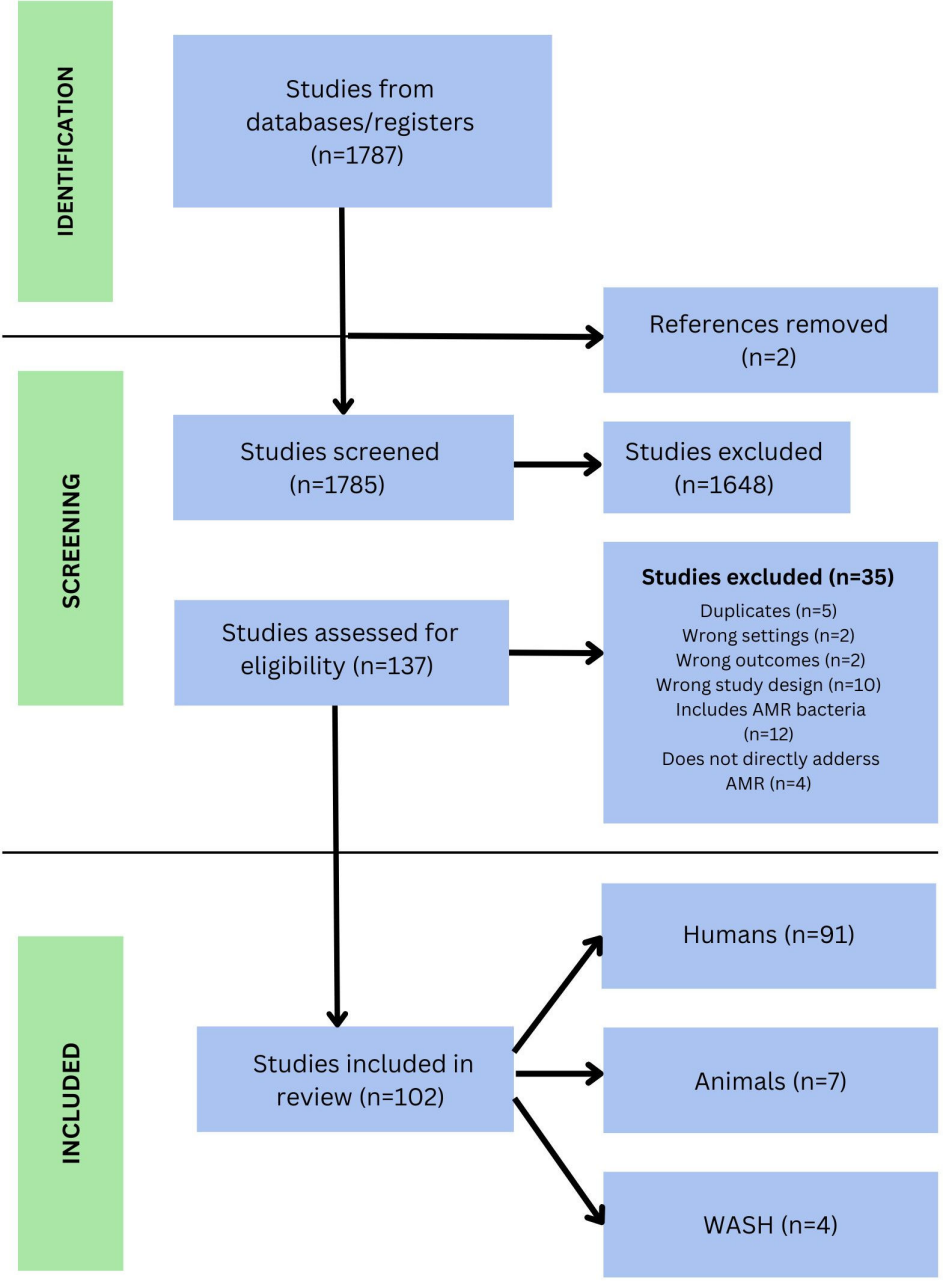
Preferred reporting items for systematic reviews and meta-analyses (PRISMA) flow diagram of the article selection process for this systematic review.

The search strategy identified a total of 1,787 articles. After removing duplicates, 1,785 studies remained for the title and abstract screening. Following the initial screening, 137 full-text studies were assessed for eligibility. Of those, 102 articles met the inclusion criteria and were included in the analysis (Fig. 1). Adopting the One Health approach, the studies were categorized into those focused on humans, animals, and the environment as outlined below.

### Humans

Ninety-one studies examined human subjects. The majority of studies (76) were empirical investigations involving human patients; 12 studies explored knowledge, attitudes, and practices (KAP) related to AMR, and three studies addressed AMR in the context of travel for medical reasons.

#### Studies on AMR in humans

**Fig 2:**
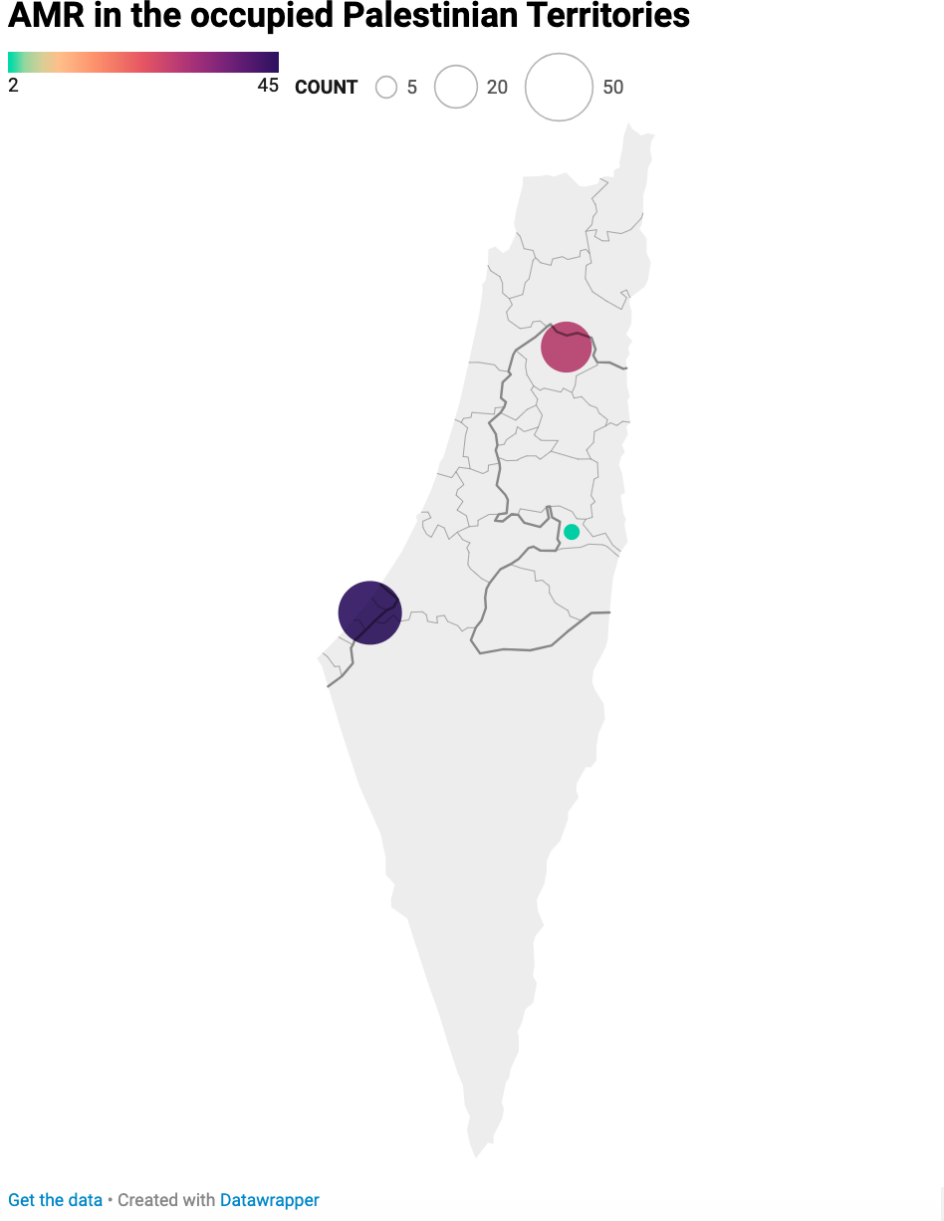
Map of occupied Palestinian Territories displaying the distribution of articles used in this review across the West Bank, Gaza Strip, and East Jerusalem.

Table S1 summarizes the characteristics of empirical studies examining antimicrobial resistance (AMR) among human patients in the oPt. All selected articles were in English and focused on Palestinian patients in the Gaza Strip (55.3%), West Bank (38.2%), and East Jerusalem (5.3%), as shown in Fig. 2. Over time, there has been a noticeable increase in the number of studies on AMR, with only four published between 1997 and 2002, compared with 25 between 2015 and 2020, as illustrated in Table S1.

Approximately half of the studies (44.7%) were conducted in hospital and inpatient settings, whereas a smaller portion (13.1%) focused on outpatient samples. The most common clinical presentation was urinary tract infection (UTI) (19.7%), followed by gastrointestinal disorders (6.6%), sepsis (5.3%), skin and wound infections (3.9%), and ear, nose, and throat infections (3.9%). Mixed clinical presentations were described over a third of the studies (35.5%), and 25% focused on bacterial colonization. The types of samples examined included urine (13 studies), feces (5 studies), blood (3 studies), and mixed specimens (53 studies).

Most studies (48) included participants of mixed ages, whereas 13 focused on children, 12 on adults, and 3 specifically on neonates and infants. The primary driver of AMR cited in the articles was the “abuse of antibiotics” (53.9%). Other suggested drivers included nosocomial and community transmission pathways, lack of national guidelines for antibiotic use, over-the-counter (OTC) availability of antibiotics, environmental contamination, and genetic mutations. The least commonly mentioned driver was “war/siege” (1.3%). A majority of the studies (63.2%) used one of the two international guidelines for measuring microbial resistance: the Clinical Laboratory Standards Institute (CLSI) guidelines (39.5%) and the National Committee for Clinical Laboratory Standards (NCCLS) guidelines (23.7%).

#### Bacteria examined within humans

The most frequently described pathogens were *Escherichia coli* (32.9%), *Staphylococcus* aureus (30.3%), *Klebsiella pneumoniae* (11.8%), *Streptococcus pneumoniae* (7.9%), *Salmonella,* and *Shigella* (3.9% each). Overall, antibiotic susceptibility testing was conducted for 47 different antibiotics. There was considerable variation in patient populations (adults vs. children, inpatients vs. outpatients), specimen types (pus, blood, urine, and stool), and clinical syndromes, including cases of bacterial colonization. Some studies included mixed populations within the same study, adding to the heterogeneity. Differences in laboratory methods for testing antibiotic resistance and reporting AMR profiles also contributed to the variability. Due to this heterogeneity, a meta-analysis of the different AMR profile frequencies was not feasible. Table S2 provides a summary of the median percentages and interquartile ranges (IQR) for the most frequently examined AMR profiles.

**Fig 3:**
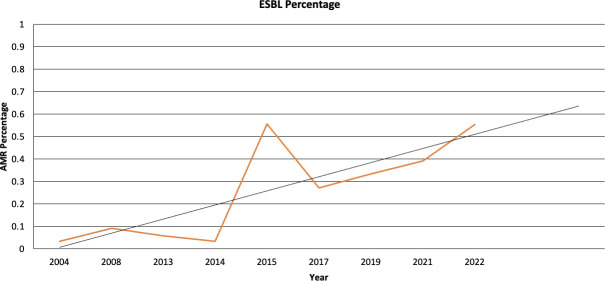
Graph displaying temporal trends in ESBL-production among *E. coli* samples analyzed within articles used for this review.

More than a quarter (27.3%) of S. *aureus* isolated were methicillin-resistant *S. aureus* (*MRSA*). Similarly, 27% of *E. coli* isolates showed extended-spectrum beta-lactamase (ESBL) profile, with 5.8% being carbapenem-resistant. Forty percent of *K. pneumoniae* isolates exhibited ESBL. A study evaluating 322 gram-negative bacillus (GNB) isolates from four referral pediatric hospitals in Gaza found an ESBL profile in 55.3% of the 170 isolated *E. coli* and 63.6% of the 66 isolated K. *pneumoniae* (63). More than half (55%) of the S. *pneumoniae* isolates were resistant to penicillin. Eight percent of the *Acinetobacter* spp. isolates were carbapenem-resistant, and a third (33%) of the *Salmonella* spp. were resistant to fluoroquinolone. Figure 3 illustrates the trend of ESBL production among *E. coli* isolates over time, showing a positive slope in the trendline.

#### Studies on knowledge, attitudes, and practices toward AMR

Twelve studies focused on the KAP related to antibiotics misuse and its connection to AMR. These studies assessed various populations, including physicians, pharmacists, patients, and parents. Table S3 summarizes the findings across different levels and stakeholders, including policies, physicians, pharmacists, patients, and social determinants of health (SDOH). The studies identified several different drivers of inappropriate antibiotic use, such as prescribing, dispensing, and purchasing behaviors. Three studies (64–66) highlighted the absence of national guidelines for antibiotic use in both surgical and medical contexts. High rates of inappropriate antibiotic prescribing by physicians for common viral infections were noted (67, 68), with one study reporting that 67.5% of prescriptions in primary healthcare settings included antibiotics (69). Issues such as omissions of frequency, dosage, and duration were common in prescription records. Moreover, many pharmacists believed that dispensing unnecessary antibiotics was harmless (64, 69). Pharmacists often encountered unnecessary antibiotic prescriptions and sold antibiotics without prescriptions in response to patient demands. One study found that 77% of pharmacists had encountered unnecessary antibiotic prescriptions, and 53.2% believed that unnecessary dispensing posed no risk to public health (64).

One study indicated that 80% of participants viewed antibiotic use favorably for common viral conditions such as upper respiratory tract infections and sore throat (65, 70), reflecting a widespread belief in the benefits of antibiotics, even when unnecessary. Another study found that individuals from higher-income households had a better understanding of appropriate antibiotic use compared with those from lower-income backgrounds (71) and that up to 70% of households hoarded antibiotics for use without medical consultation and shared them with family members and neighbors (65). Common reasons for self-medication included a lack of awareness about the side effects of antibiotics and AMR, long waiting times for medical appointments, and the financial burden of seeing a doctor. Patients also cited fears of delayed recovery without antibiotics and concerns about missing workdays.

One study directly linked political factors, such as curfews and checkpoints, to the tendency to hoard antibiotics at home for emergencies (65). Another study discussed the siege on the Gaza Strip, which disrupted the supply chain of antibiotics. This disruption led to the use of advanced broad-spectrum antibiotics before their expiration and the hoarding of antibiotics by patients, further promoting AMR (72).

#### Traveling for medical treatment to Israel

The review identified three studies examining antimicrobial resistance (AMR) among Palestinian patients who received medical treatment in Israeli hospitals. The clinical conditions of these patients varied. Two studies focused on a large number of isolates from patients with mixed clinical diagnoses, whereas one specifically examined patients infected with OXA-48-producing Enterobacteriaceae (73–75). All three studies were conducted in inpatient hospital settings. Specimen collection methods included rectal swabs, rectal swabs combined with blood samples, and mixed samples of unspecified types. The most commonly assessed bacteria were Enterobacteriaceae and *K. pneumoniae*, both of which were analyzed in two of the three studies (73, 74).

One study investigated different AMR patterns by examining 1,653 Palestinian patients and 108,783 Israeli patients, finding that Palestinian patients had significantly higher carriage rates of AMR bacteria compared with their Israeli counterparts (75). Specifically, carbapenem-resistant Enterobacterales (CRE) were detected in 1.9% of Palestinian patients, compared with 0.7% of Israeli patients (75). Vancomycin-resistant *Enterococcus* (VRE) was identified in 1.5% of Palestinian patients versus 0.6% of Israeli patients (75). Methicillin-resistant *S. aureus* (MRSA) was found in 3.5% of Palestinian patients, compared with 1.1% of Israeli patients (75). Carbapenem-resistant *Acinetobacter baumannii* was present in 4.4% of Palestinian isolates, compared to 0.7% among Israeli patients (75).

Another study focused on carbapenemase-producing Enterobacteriaceae (CPE) in an Israeli post-acute care hospital (74). This study found that all four OXA-48 CPE cases (out of 127 CPE samples) were acquired either directly or indirectly from patients arriving from the Palestinian Authority or Syria (74). A separate study specifically examining OXA-48-producing Enterobacteriaceae in Israeli hospitals identified four cases, all involving medical tourists, with two of the cases involving Palestinian patients (73).

### Studies on AMR in animals

Our review identified seven studies that evaluated antimicrobial resistance (AMR) in animals and animal-based products and foods (Table S4). The studies included chickens (*n* = 4), cows (*n* = 2), and a combination of cows and goats (*n* = 1). Only one study focused on animals with a clinical condition (cows with mastitis); the remaining studies were conducted for surveillance or carriage. Two studies were conducted in Gaza, both examining AMR in chickens, whereas five studies in the West Bank included chickens, cows, and goats. All studies utilized disk diffusion methods to identify AMR, with results reported according to Clinical Laboratory Standards Institute (CLSI) or National Committee for Clinical Laboratory Standards (NCCLS) guidelines, although the specific methods varied across studies (76–82).

Aligned with the One Health perspective, one study assessed bacteria from cattle products, humans, and environmental surfaces on a cattle farm (77). Another study analyzed both animal and human samples, testing for *Salmonella* in poultry and humans (78), whereas a third study examined both environmental samples and animals, focusing on water and poultry products across different farms (79). Across all studies, 87 samples were collected from humans, 972 from animals and animal products, and 45 from environmental surfaces. Of the 972 animal samples, 60.0% (583 samples) were found to be infected with bacteria of interest.

There was a lack of uniformity in antibiotic testing across the studies, with different sets of antibiotics tested. However, tetracycline was assessed in six of seven studies, showing resistance rates ranging from 40.9% to 100% (81, 82). In poultry, *E. coli* showed 100% resistance to tetracycline, whereas resistance in cattle product samples was 76.9% (80, 82). Nalidixic acid resistance was tested in two studies, revealing a wide range of resistance from 59% in *Salmonella* samples from poultry to 94.8% in *S. aureus* samples from cattle-based products (78, 80). Meropenem resistance also varied significantly, ranging from 3% in bacteria from poultry samples to 76.9% in cattle product samples (79, 80). Carbapenem resistance was observed at 36% in poultry samples (79). Up to 45.6% of *S. aureus* isolates from cow milk were identified as MRSA (80). Conversely, ceftriaxone resistance remained at or below 5% in all isolates tested across two studies (77, 78).

The most commonly examined bacterium was *E. coli* (in three of seven studies). High rates of AMR were frequently attributed to the lack of regulation surrounding the use of antibiotics in animals (six of seven studies). One study specifically mentioned inadequate hygiene and sterilization practices during breeding and slaughtering processes, which could exacerbate the transfer of antibiotic-resistant genes between and within species (82).

### Studies on AMR in WASH

Four studies investigating AMR in water sources were included in this review (83–86). Three of these studies were conducted in the Gaza Strip, and one was conducted in the West Bank (83, 85, 86). Among the Gaza-based studies, one focused on coastal water samples, whereas the other two examined water from hospital settings. The study from the West Bank investigated irrigation water (84). Across all studies, a total of 871 water samples were collected, with 597 of these samples found to be contaminated with bacteria. The contamination rates ranged from 34.1% to 100%, with an average contamination rate of 65.5% (83, 85).

The most commonly tested bacteria were Enterobacteriaceae, *Pseudomonas*, and *S. aureus*, which were examined in three of the four studies (83–85). Two studies conducted in Gaza tested Enterobacteriaceae for resistance to imipenem, finding resistance rates of approximately 66.7% and 57.6%, respectively (83, 85). In contrast, the study conducted in the West Bank found no resistance, with 100% of Enterobacteriaceae isolates susceptible to imipenem (84).

Tap water and wastewater samples from hospitals in Gaza revealed that 25% of Enterobacteriaceae isolates were OXA-producing (83). Another study from Gaza reported that 51.9% of the water samples were contaminated with coliform bacteria, and 5.6% of these isolates were ESBL-producing, with imipenem resistance at 66.7% (86). A study examining coastal water samples in Gaza found that all (100%) samples were contaminated with bacteria, with the highest resistance rates against penicillin (75.1%) and ampicillin (76.7%) (85). Among Enterobacteriaceae isolates, 96.6% were identified as multidrug-resistant (MDR), with an overall MDR rate of 85% across all bacterial isolates (85).

The study analyzing gray water used in agriculture systems in the West Bank found that 76% of water samples were contaminated with bacteria, including *E. coli*, *Klebsiella* sp., and other Enterobacteriaceae (84). The highest resistance observed was to ampicillin (69.3% of isolates), whereas the lowest resistance was to cefazolin (7.9% of isolates). Additionally, 7.9% of isolates from gray water in the West Bank were MDR (84).

None of the four studies explicitly mentioned the impact of ongoing armed conflict on WASH in the occupied Palestinian territories (oPt). However, one article suggested that the presence of MDR bacteria in coastal waters around Gaza could be attributed to the discharge of waste from animal farms and untreated sewage from hospitals (85).

## DISCUSSION

### AMR in the context of one health and global health

Following the One Health approach to AMR surveillance (22), this study explored knowledge production about AMR in humans, animals, and the environment within the the Palestinian territories occupied by Israel in 1967 (oPt). Although AMR poses a growing threat to global and planetary health, it is an even more pressing issue in regions affected by war, conflict, and settler colonization. Such areas, including the oPt, often lack the necessary resources to effectively combat the spread of AMR. The large number of refugees originating from war-affected regions further exacerbates the global spread of AMR. In the oPt, the situation is intensified by ongoing military occupation and various political, social, and environmental health determinants (26), which subject communities to chronic war-like conditions punctuated by periods of heightened military activity (87).

### Prevalence and patterns of AMR

Several studies indicate that AMR is widespread in the oPt. The median percentage of extended-spectrum beta-lactamase (ESBL) production was 27% for *E. coli* and 40% for *K. pneumoniae*, with some studies reporting ESBL rates up to 50% and 60%, respectively (63). These figures are comparable with those observed in other low- and middle-income countries (LMICs) like Nepal (34%) (88) and Kenya (44%) (89), as well as conflict-affected regions like Syria (6) and other Middle Eastern countries (9, 90). However, these rates are significantly higher than those reported in high-income countries such as the United States (7%–15%) (91).

The median MRSA rate in the review was 27.3%, with some studies reporting rates as high as 80% (92). These MRSA rates are similar to those found in other LMICs (93, 94) and lower than some other Middle Eastern countries, where median MRSA rates reach 45% (9). Alarmingly, 42.1% of isolates from healthcare workers were positive for MRSA (95), a finding comparable with reports from Syria (96).

Figure 3 shows a time-related increase in AMR, particularly in ESBL production among *E. coli*. Although studies used different sampling and laboratory methods, this trend highlights the growing inability to manage and contain AMR as a public health threat.

### Factors contributing to AMR in the oPt

AMR is a global public health crisis that has been escalating over the past decades (1, 97). It is not solely driven by the overuse of antibiotics. Multiple studies link AMR to inadequate water, sanitation, and hygiene (WASH) infrastructure, poverty (98), and weak vaccination programs in the Global South, which lead to environmental contamination and a higher burden of infectious diseases, necessitating antibiotic use (99). The changing nature of war in the past decades, affecting more civilian and urban spaces, has increasingly been recognized as a driver of AMR due to healthcare system collapse and the lack of effective AMR monitoring or treatment (7). In the oPt, these drivers are intensified. Prolonged settler colonization and war-like conditions not only lead to impoverishment and de-development but also to the destruction of WASH and medical infrastructure (44, 56) and the hindering of vaccination programs (100). Unregulated humanitarian missions and a continuous fear of drug shortages lead to antibiotic hoarding and misuse. These factors, combined with the ongoing dismantling of the state in general and its ability to regulate medical and pharmaceutical practices, impede efforts to address the AMR crisis effectively.

### Socio-political context and AMR research gaps

An important finding from this review is the lack of engagement with the socio-political context in AMR research. For Palestinians living under settler colonization, military occupation, and chronic war-like conditions, the right to health is compromised in multiple ways. The study of AMR provides us with another lens of understanding the complexities in which the right to health is compromised: access to appropriate antibiotics, standardized proper laboratory testing of AMR, uniform AMR prescription protocols, and the ability to carry out proper wound management in war times, often infected with AMR bacteria. The lack of available appropriate antibiotics and antibiotic anarchy both harm the right to health of Palestinians. Although studies of AMR in conflict-affected countries in the Arab region, such as Yemen (12), Syria (6), Sudan (101), and Iraq (7), often contextualize their findings within the scope of war and healthcare system destruction (9), research in the oPt rarely addresses the sociopolitical realities of war, blockade, and military occupation. Most articles attribute the high AMR rates to general shortcomings in antibiotic stewardship and local practices, such as over-prescription by doctors and self-medication by patients, neglecting upstream structural factors that could contextualize these behaviors.

Despite increasing calls within public health scholarship to acknowledge structural and political determinants of health (25, 30, 31, 102, 103), few studies discuss the political context (104) as a key factor weakening the healthcare system and hampering the enforcement of national antibiotic policies. Physicians and other healthcare providers in this context may lack exposure to critical public health theories and frameworks, which limits their ability to engage with the social and political determinants of health (105). This omission might be due to self-censorship or censorship by academic journals (106–108) regarding the political reality of Palestinians. Another explanation is the normalized state of war and settler colonization in Palestine, which has persisted for over seven decades (25), making war a pervasive and often overlooked aspect of health-related research (109).

### Specific concerns related to WASH infrastructure and environmental health

The interconnected nature of human, animal, and environmental health is particularly evident during periods of wars. Repeated military assaults on the Gaza Strip have led to the destruction of WASH systems and sewage treatment facilities, exacerbating public health crises (110, 111). The attacks on Palestinian sewage infrastructure contribute to the contamination of drinking water, coastal sea pollution, and the spread of AMR organisms (111). Abushomar et al. found that among ESBL genes identified in Enterobacteriaceae in tap water and wastewater samples from Gaza hospitals, 25% were OXA-positive, compared with 2.5% reported in Israeli hospitals (83, 112). Despite this, few studies explicitly discuss the impact of warfare on water sanitation measures.

### Impact of recent escalations and broader implications

While this paper was being reviewed, the Gaza Strip was subjected to one of the deadliest episodes of warfare following the 7 October attacks by Hamas. This war, deemed a genocide by leading human rights organizations (49, 113, 114), resulted in the displacement of nearly two million Palestinians and extensive destruction of healthcare and WASH infrastructure (115). The combined effects of overcrowded living conditions, lack of clean water, electricity outages, and damaged healthcare facilities are a public health crisis with potential severe AMR outbreaks (115). The current situation mirrors past wars, such as the Iraq War, where returning soldiers carried AMR pathogens (7). Several reports of Israeli soldiers dying from AMR are emerging (116–118).

The destruction of much of Gaza’s healthcare system and laboratories hampers the collection of reliable AMR data. This new reality brings into question the applicability of earlier findings and suggests that AMR could become a “silent threat” in the ongoing genocidal war (115). A severe depletion of nearly all medical resources including antibiotics has led to conditions such as diabetic foot conditions—typically treatable with antibiotics—resulting in foot amputations (115, 119). Moreover, patients with deep wounds are often unable to receive necessary surgical interventions as needed, leading to infections that require antibiotics, but are frequently inaccessible due to shortages in medical supplies (97, 119). The Israeli genocidal war has led to systemic destruction of the healthcare system (120), making antibiotic stewardship “an unattainable luxury” (115, 119). Physicians completing medical missions in Gaza discussed the immense challenges in properly prescribing and safely accessing antibiotics due to the Israeli siege and developed their own guidelines for the new circumstances created by the genocide (121).

### Recommendations

Addressing AMR in the oPt and similar war-affected regions necessitates a comprehensive One Health approach that integrates human, animal, environmental health, and the infrastructures connecting them. It is essential to understand and respond to the socio-political context influencing health outcomes. Effective AMR management requires national, regional, and international collaboration for both treatment and surveillance. However, in a setting marked by intense warfare, systematic destruction of health and WASH facilities, attacks on healthcare workers, and ongoing civilian casualties, these ideal recommendations often become impractical. Addressing and dismantling the upstream drivers of war, settler colonization, military occupation, and siege are critical for improving Palestinian health and healthcare (122, 123), including managing infectious diseases and AMR. Nonetheless, pragmatic and actionable steps are necessary to urgently address AMR in the oPt. Below are specific recommendations for the Gaza Strip following the unprecedented 2023–2024 genocidal war, along with general recommendations for the broader oPt.

#### Recommendations specific for the Gaza Strip

##### Immediate humanitarian actions

The top priority should be ending the warfare and siege, ensuring the unconditional delivery of antibiotics and medical equipment to the Gaza Strip, reconstructing WASH infrastructure, treating infectious diseases empirically, and facilitating proper wound care, sterile environments for invasive procedures, and effective infection control practices (56, 124).

##### Re-establishing vaccination programs

Despite limited resources, vaccination rates among Palestinians in the oPt have been higher than in other LMICs (125). However, the recent destruction of healthcare infrastructure in Gaza has severely impacted the vaccination program. It is crucial to restart these programs, particularly for children who missed vaccinations due to the war, to reduce the burden of infectious diseases, minimize antibiotic use, and curb the development of AMR.

##### Rebuilding laboratory and surveillance capabilities

Efforts should focus on rebuilding the laboratory sector and conducting mass examinations of specimens for AMR in both inpatient and outpatient settings using WHO guidelines (126). Establishing a robust national surveillance system for AMR, aligned with the WHO Global Action Plan (GAP), is also essential (127, 128).

##### Developing best practice guidelines

A team of infectious disease and AMR experts should be established to develop and regularly update best practice guidelines for treating common infectious diseases, including war-related injuries in post-war Gaza (128).

##### Control over-the-counter antibiotic dispensation

In the later stages of recovery, measures enforced by the ministry of health and professional unions should be implemented to control the over-the-counter sale of antibiotics across the Gaza Strip.

### Recommendation for the oPt

#### Institutional stewardship

Strengthen collaborations between physicians and pharmacists to regulate antibiotic prescriptions, reduce antibiotic use, and introduce facility-wide restrictions on broad-spectrum antibiotics. Implement additional reviews for complex cases and new paperwork to track and manage antibiotic prescriptions (129, 130).

#### Enhance diagnostic testing accessibility

Ensure that point-of-care diagnostic testing is widely available. Improved diagnostic capabilities will enable more targeted treatment guidelines and provide better assessments of disease prevalence and AMR magnitude (131).

#### Addressing patient fears

Healthcare providers in the oPt should actively address patients' concerns about antibiotic shortages during medical consultations. Given the frequent disruptions to the supply of vital drugs due to warfare, clinicians must engage with patients to alleviate fears and discourage hoarding and misuse of antibiotics. Clinicians working through foreign aid organizations should prioritize understanding and addressing the legitimate concerns of patients regarding access to antibiotics and medical care during blockades or escalated violence.

#### Developing AMR reporting guidelines

Healthcare providers in the oPt should create guidelines that adhere to international standards for AMR study and reporting, as defined by the World Health Organization in the Global Antimicrobial Surveillance System (GLASS) guidelines (127). Comprehensive and well-documented AMR stewardship is necessary to grasp the extent of the problem and implement effective countermeasures.

#### Establishing a national antibiotic policy

A well-regulated national antibiotic policy must be developed in the oPt. Training programs for clinicians should emphasize the risks of antibiotic misuse, AMR development, and strategies to combat these issues (132, 133).

#### Contextualizing research within the socio-political framework

Future research on AMR and health outcomes in Palestine should be framed within a socio-political context. The historical and ongoing warfare and settler colonialism, as well as political instability, should be recognized as significant structural determinants of health. These factors influence everything from patient behaviors to the regulation of antibiotic prescriptions, sales, and import/export processes. Acknowledging these socio-political determinants will provide a more comprehensive understanding of AMR dynamics in the region (26, 32).

### Limitations

Our review has several limitations that need to be considered when assessing our findings. First, we only included papers in English and not in Arabic as not all involved researchers are fluent in Arabic. Second, we did not do a quality assessment of the papers included, as all studies were observational studies and not interventional, and we were more interested in mapping published literature rather than examining the quality of published studies. Furthermore, due to the lack of standardization on investigating and reporting of AMR in the oPt, the studies’ data differed greatly in relation to the bacteria and antibiotics tested. Resistance to more 47 antibiotics was reported; thus, the results extracted from studies focused on the bacteria most thoroughly investigated in each respective study and the most reported AMR profiles across studies. This is a serious limitation that prevents us from conducting meta-analysis. Although the fragmented nature of the health services in the oPt contributes to this lack of standardization, scholars from other parts of the world have faced a similar limitation while conducting reviews on AMR (9, 134–136). Despite these limitations, the consistency of our findings with other war-affected regions in the Middle East supports the validity of our results.

## Supplementary Material

Reviewer comments

## SUPPLEMENTAL MATERIAL

Supplemental tables msystems.01679-24-s0001.docx Tables S1 to S4.

Supplemental material msystems.01679-24-s0002.docx Sample search strategy.

OPEN PEER REVIEW reviewer-comments.pdf An accounting of the reviewer comments and feedback.

ASM does not own the copyrights to Supplemental Material that may be linked to, or accessed through, an article. The authors have granted ASM a non-exclusive, world-wide license to publish the Supplemental Material files. Please contact the corresponding author directly for reuse.
