# Supplementary material for: Antimicrobial resistance in a protracted war setting: a review of the literature from Palestine
Source: mSystems. 2025 May 21;10(6):e01679-24. doi: 10.1128/msystems.01679-24 (PMC12172458; doi:10.1128/msystems.01679-24)
Supplement: Supplemental tables — Tables S1 to S4. [file msystems.01679-24-s0001.docx]

Table 1 – Characteristics of the empirical studies on AMR in human subjects in the oPt

| **Study Characteristics** | **Number (%)**  **Total= 76** | **References** |
| --- | --- | --- |
| **YEAR OF PUBLICATION** |  |  |
| 1997-2002 | 4 (5.3%) | (1–4) |
| 2003-2008 | 15 (19.7%) | (5–19) |
| 2009-2014 | 20 (26.3%) | (20–39) |
| 2015-2020 | 25 (32.9%) |  |
| 2021-2023 | 12 (15.8%) | (65–76) |
| **LOCATION STUDY** |  |  |
| Gaza | 42 (55.3%) |  |
| West Bank | 29 (38.2%) | (2, 17–22, 24, 28, 29, 31, 32, 34, 36, 41, 42, 44, 45, 47, 52–56, 60, 63, 66, 71, 75) |
| East Jerusalem | 4 (5.3%) | (3, 38, 50, 68) |
| Mixed | 1 (1.31%) | (35) |
| **PATIENT CHARACTERISTICS** |  |  |
| Inpatient | 34 (44.7%) | (1, 3, 7, 9, 14, 16, 18, 19, 21, 28, 30, 38, 40-42, 44- 46, 49-52, 54, 55, 59, 60, 63-66, 68, 70, 73, 76) |
| Outpatient | 10 (13.1%) | (4, 6, 15, 22, 23, 26, 31, 36, 37, 58) |
| Mixed | 23 (30.3%) | (2, 5, 8, 10, 11, 12, 17, 20, 29, 33, 35, 39, 47, 48, 53, 56, 61, 62, 67, 69, 72, 74, 75) |
| Other (environmental sample, healthy community members, healthcare workers) | 9 (11.8%) | (13, 24, 25, 27, 32, 34, 43 57, 71) |
| **AGE GROUP** |  |  |
| Children | 13 (17.1%) | (1, 2, 9, 12, 27, 28, 31, 34, 37, 38, 68, 74, 76) |
| Adults | 12 (15.8%) | (4, 6, 15, 24, 32, 43, 45, 57, 58, 60, 61, 66) |
| Neonates and infant | 3 (3.9%) | (30, 50, 73) |
| Mixed | 48 (63.2%) | (3, 5, 7, 8, 10, 11, 13, 14, 16-23, 25, 26, 29, 33, 35, 36, 39- 42, 44, 46- 49, 51-56, 59, 62- 65, 67, 69-72, 75) |
| **CLINICAL SYNDROME** |  |  |
| Urinary tract infection | 15 (19.7%) | (4, 6, 10, 15, 19, 21, 22, 36, 38, 46, 50, 53, 61, 65, 74) |
| Gastrointestinal diseases | 5 (6.6%) | (9, 12, 17, 23, 60) |
| Sepsis and blood stream infections | 4 (5.3%) | (28, 30, 45, 73) |
| Skin and wound infection | 3 (3.9%) | (33, 51, 54) |
| ENT infection | 3 (3.9%) | (1, 2, 37) |
| Colonization | 19 (25%) | (13, 24-27, 32, 34, 39, 41-44, 49, 57, 58, 63, 66, 69, 71) |
| Mixed | 27 (35.5%) | (3, 5, 7, 8, 11, 14, 16, 18, 20, 29, 31, 35, 40, 47, 48, 52, 55, 56, 59, 62, 64, 67, 68, 70, 72, 75, 76) |
| **TYPE OF SPECIMEN** |  |  |
| Urine | 13 (17.1%) | (4, 6, 10, 15, 19, 21, 22, 38, 46, 50, 53, 61, 74) |
| Blood | 3 (3.9%) | (28, 30, 73) |
| Pus | 1 (1.3%) | (8) |
| Tissue | 1 (1.3%) | (3) |
| Feces | 5 (6.6%) | (9, 12, 13, 23, 39) |
| Mixed | 53 (69.7%) | (1, 2, 5, 7, 11, 14, 16-18, 20, 24, 25-27, 29, 31-37, 40- 45, 47-49, 51, 52, 54-60, 62- 72, 75, 76) |
| **SUGGESTED DRIVERS OF AMR** |  |  |
| Abuse of antibiotics | 41 (53.9%) | (2-4, 8, 10, 11, 13, 15, 17, 19-21, 24, 25, 27, 30, 33, 34, 37, 39, 40, 43, 47, 50, 51, 54-59, 61, 64-68, 70, 72, 75, 76) |
| Community pathways | 18 (23.7%) | (6, 11, 25, 29, 30, 35, 38-40, 43, 45, 47, 50- 52, 54, 70, 72) |
| Nosocomial (including overcrowding in hospitals) | 19 (25%) | (6, 11, 25, 29, 30, 35, 38- 40, 43, 45, 47, 50-52, 54, 70-72) |
| Lack of national guidelines for antibiotic use and dispensation | 15 (19.7%) | (10, 24, 27, 34, 40, 47, 51, 54, 57, 63, 64, 70, 72, 75, 76) |
| OTC availability of antibiotics | 12 (15.8%) | (2, 8, 11, 16, 27, 37, 38, 47, 54, 59, 66, 72) |
| Contamination from environment  (Agriculture, overcrowding and sewage water) | 9 (11.8%) | (4, 13, 17, 23, 26, 39, 54, 57, 64) |
| Genetic mutations | 6 (7.9%) | (26, 29, 34, 48, 49, 59) |
| War / Siege | 1 (1.3%) | (23) |

Table 2 – Proportion of AMR for different GLASS pathogens in the oPt.

| GLASS PATHOGEN | Resistance Profile of public health importance | Overall percentage median resistance  (IQR) | Number of articles | References |
| --- | --- | --- | --- | --- |
| S. aureus | MRSA | 27.3 (9-56.3) | 17 | (7, 18, 20, 24, 26, 32, 33, 37, 40, 41, 43, 49, 54, 57, 63, 68, 71) |
|  | VRSA | 0.3 (0-1.5) | 6 | (3, 5, 7, 29, 43, 73) |
| E. coli | ESBL- producing | 27  (5.8- 39.1) | 9 | (6, 11, 36, 38, 46, 51, 53, 59, 72) |
|  | Carbapenem- resistant | 5.8  (0-16) | 14 | (11, 19, 21, 46, 48, 51, 54, 59, 61, 65, 70, 72, 73, 76) |
| Acinetobacter spp. | Carbapenem- resistant | 8 (7.5-10) | 5 | (8, 25, 30, 35, 73) |
| K. pneumoniae | ESBL- producing. | 40  (34-57.1) | 7 | (11, 46, 51, 53, 59, 72, 76) |
|  | Carbapenem-resistant |  | 1 | (48) |
| S. pneumoniae | Penicillin non-susceptible | 55  (43.5-66.5) | 6 | (1, 2, 5, 27, 28, 34) |
| Salmonella spp. | Fluoroquinolone- resistant | 33  (22.5-33) | 3 | (9, 12, 23) |
| Shigella spp. | Fluoroquinolone- resistant | 4  (2-27) | 3 | (9, 12, 23) |

Table 3 – Summary of suggested drivers of AMR in studies on knowledge, attitudes and practices of patients

| **GUIDELINE TYPE** | **References** |
| --- | --- |
| **POLICY** |  |
| Lack of antibiotic dispensation guidelines for physicians and pharmacies | (78–80) |
| Lack of treatment guidelines for medical and surgical conditions | (81, 82) |
| Lack of continuous medical education and training for medical staff | (78) |
| **PHYSICIANS** |  |
| High number of unnecessary prescriptions | (78, 79, 82–84) |
| Lack of training on proper antibiotic use | (81) |
| Abuse of broad-spectrum antibiotic for extended times | (82) |
| **PHARMACY** |  |
| Dispensing antibiotics without prescription | (78–80) |
| **PATIENTS** |  |
| Self-medication | (79, 81, 85) |
| High demand of antibiotics for sore threat, upper respiratory infections, and other viral infections | (78, 79, 81, 83, 85, 86) |
| Sharing antibiotics and using leftover antibiotics | (87) |
| Drug-hoarding behavior due to fear of unexpected future shortage | (88) |
| **SOCIAL DETERMINANTS OF HEALTH** |  |
| Political causes (i.e. siege, lack of resource coordination between West Bank and Gaza) | (88) |
| Improvising between available antibiotics and those about to expire - unstable drug supply chain | (88) |
| Political conflict, curfews and checkpoints inciting fear and having antibiotics in the house just in case. | (79) |
| Inconvenient access to MOH clinics leading to patients’ self-medication with antibiotics | (80) |
| Time and monetary cost of going to the doctor | (85) |

Table 4 – Characteristics of studies on AMR in animals

| **AMR IN ANIMALS: STUDY CHARACTERISTICS** | NUMBER  TOTAL = 7 | References |
| --- | --- | --- |
| **ANIMAL TYPE** |  |  |
| Chicken | 4/7 | (31, 89–91) |
| Cows and goats | 3/7 | (92–94) |
| **LOCATION** |  |  |
| West Bank | 5/7 | (31, 91–94) |
| Gaza | 2/7 | (89, 90) |
| **Bacteria examined** | Percentage of AMR |  |
| VRE | 44% | (89) |
| Shiga-toxin producing E. coli | 50% | (92) |
| Carbapenem-resistant gram-negative bacteria | 36% | (90) |
| MRSA | 45.6% | (93) |
| Gentamycin-resistant E. coli | 50% | (91) |

**Bibliography:**

1. El-Astal Z, Khamis N, Peled N, Dagan R, Yagupsky P. 1997. Antimicrobial resistance and typing of pneumococci in Gaza Strip children. The Pediatric infectious disease journal 16:905–907.

2. Adwan K, Abu-Hasan N, Hamdan A, Al-Khalili S. 1999. High incidence of penicillin resistance amongst clinical isolates of Streptococcus pneumoniae in northern Palestine. Journal of medical microbiology 48:1107–1110.

3. Essawi T, Na’was T, Hawwari A, Wadi S, Doudin A, Fattom AI. 1998. Molecular, antibiogram and serological typing of Staphylococcus aureus isolates recovered from Al-Makased Hospital in East Jerusalem. Tropical medicine & international health : TM & IH 3:576–583.

4. Astal Z, El-Manama A, Sharif FA. 2002. Antibiotic resistance of bacteria associated with community-acquired urinary tract infections in the southern area of the Gaza Strip. Journal of chemotherapy 14:259–264.

5. El-Astal Z. Bacterial pathogenes and their antimicrobial susceptibility in Gaza Strip, Palestine. Pak J Med Sci 20:365–370.

6. Astal Z, Sharif FA, Abdallah SA, Fahd MI. 2004. Extended spectrum beta-lactamases in Eschericia coli isolated from community-acquired urinary tract infections in the Gaza Strip, Palestine. Annals of Saudi medicine 24:55–57.

7. Abu Hujier NS, Sharif FA. 2008. Detection of methicillin-resistant Staphylococcus aureus in nosocomial infections in Gaza Strip. African Journal of Microbiology Research 235–241.

8. Elmanama AA. 2006. Antimicrobial Resistance of Acinetobacter spp. Isolated from Pus Specimens from AL-Shifa Hospital, Gaza, Palestine. Al-Aqsa University Journal (Natural Sciences Series) 10:59–68.

9. Abu Elamreen FH, Abed AA, Sharif FA. 2007. Detection and identification of bacterial enteropathogens by polymerase chain reaction and conventional techniques in childhood acute gastroenteritis in Gaza, Palestine. International Journal of Infectious Diseases 11:501–507.

10. Elmanama AA, Elaiwa NM, El-Ottol AEY, Abu-Elamreen FH. 2006. Antibiotic resistance of uropathogens isolated from Al-Shifa hospital in Gaza Strip in 2002. Journal of chemotherapy 18:298–302.

11. El Astal ZY, Ramadan. H. 2008. Occurrence of extended-spectrum beta-lactamases in isolates of Klebsiella pneumoniae and Escherichia coli. International journal of integrative biology 2:122.

12. Abu Elamreen FH, Sharif FA, Deeb JE. 2008. Isolation and antibiotic susceptibility of Salmonella and Shigella strains isolated from children in Gaza, Palestine from 1999 to 2006. Journal of Gastroenterology and Hepatology 23:e330–e333.

13. Elmanama AA. 2008. Vancomycin Resistant Enterococci (VRE) among Non –Hospitalized Individuals in Gaza City, Palestine. Journal of Al Azhar University-Gaza (Natural Sciences) 10.

14. Al Jarousha, Abdel-Moti K., Afifi AM. 2008. Prevalence of Multidrug Resistant Enterococci in Nosocomial Infection In Gaza Strip. Journal of Alaqsa University 12:15–24.

15. El Astal Z. 2005. Increasing ciprofloxacin resistance among prevalent urinary tract bacterial isolates in Gaza Strip, Palestine. Journal of biomedicine & biotechnology 2005:238–41.

16. Astal Z. 2004. Susceptibility patterns in Pseudomonas aeruginosa causing nosocomial infections. Journal of chemotherapy (Florence, Italy) 16:264–268.

17. Ghaleb Mohammed A, NaEl Sudqi AH, Kamel Mohammed A, Naser Rushdi J, Marwan Mohammed B. 2004. Antimicrobial resistance of Shiga toxin-producing Escherichia coli O157 isolates from Northern Palestine. Emirates Medical Journal 249–250.

18. Adwan K, Abu-Hasan N, Adwan G, Jarrar N, Abu-Shanab B, Abu-Zant A. 2005. Nosocomial infection caused by methicillin-resistant Staphylococcus aureus in Palestine. Microbial drug resistance 11:75–77.

19. Adwan K, Abu-Hasan N, Adwan G, Jarrar N, Abu-Shanab B, Al-Masri M. 2004. Molecular epidemiology of antibiotic-resistant Escherichia coli isolated from hospitalized patients with urinary tract infections in Northern Palestine. Polish journal of microbiology 53:23–26.

20. Adwan G, Abu-Shanab B, Odeh M. 2009. Emergence of vancomycin-intermediate resistant Staphylococcus aureus in north of Palestine. Asian Pacific Journal of Tropical Medicine 2:44–48.

21. Adwan K, Jarrar N, Abu-Hijleh A, Adwan G, Awwad E. 2014. Molecular characterization of Escherichia coli isolates from patients with urinary tract infections in Palestine. Journal of Medical Microbiology 63:229–234.

22. S. Abu Taha A, M. Sweileh W. 2011. Antibiotic Resistance of Bacterial Strains Isolated from Patients with Community-Acquired Urinary Tract Infections: An Exploratory Study in Palestine. Current Clinical Pharmacology 6:304–307.

23. Elmanama A, Abdelateef N. 2012. Antimicrobial Resistance of Enteric Pathogens Isolated from Acute Gastroenteritis Patients in Gaza strip, Palestine. The International Arabic Journal of Antimicrobial Agents 2:13.

24. Adwan K, Jarrar N, Abu-Hijleh A, Adwan G, Awwad E, Salameh Y. 2013. Molecular analysis and susceptibility patterns of methicillin-resistant Staphylococcus aureus strains causing community- and health care-associated infections in the northern region of Palestine. American journal of infection control 41:195–198.

25. Laham NA, Al Laham NA. 2012. Distribution and Antimicrobial Resistance Pattern of Bacteria Isolated from Operation Theaters at Gaza Strip. Journal of Al Azhar University-Gaza (Natural Sciences) 14:19–34.

26. Biber A, Abuelaish I, Rahav G, Raz M, Cohen L, Valinsky L, Taran D, Goral A, Elhamdany A, Regev-Yochay G. 2012. A typical hospital-acquired methicillin-resistant Staphylococcus aureus clone is widespread in the community in the Gaza strip. PloS one 7.

27. Regev-Yochay G, Abullaish I, Malley R, Shainberg B, Varon M, Roytman Y, Ziv A, Goral A, Elhamdany A, Rahav G, Raz M. 2012. Streptococcus pneumoniae Carriage in the Gaza Strip. PLOS ONE 7:e35061.

28. Kattan R, Abu Rayyan A, Zheiman I, Idkeidek S, Baraghithi S, Rishmawi N, Turkuman S, Abu-Diab A, Ghneim R, Zoughbi M, Dauodi R, Ghneim R, Issa AER, Siryani I, Al Qas R, Liddawi R, Khamash H, Kanaan M, Marzouqa H, Hindiyeh MY. 2011. Serotype distribution and drug resistance in Streptococcus pneumoniae, Palestinian Territories. Emerging infectious diseases 17:94–96.

29. Kaibni MH, Farraj MA, Adwan K, Essawi TA. 2009. Community-acquired meticillin-resistant Staphylococcus aureus in Palestine. Journal of medical microbiology 58:644–647.

30. Al Jarousha AMK, Jadba AHNE, Afifi ASA, Qouqa IAE. 2009. Nosocomial multidrug-resistant Acinetobacter baumannii in the neonatal intensive care unit in Gaza City, Palestine. International Journal of Infectious Diseases 13:623–628.

31. Farraj M, Al-Dawodi R, Farraj MA. 2012. Antimicrobial resistance in non-typhi Salmonella enterica isolated from humans and poultry in Palestine https://doi.org/10.3855/jidc.1167.

32. Romel Fawzi Attili R. 2013. Prevalence and Molecular Typing of Methicillin Resistant Staphylococcus aureus among Veterinary Doctors in Palestine. Al-Quds University.

33. Elmanama AA, Laham NAA, Tayh GA. 2013. Antimicrobial susceptibility of bacterial isolates from burn units in Gaza. Burns : journal of the International Society for Burn Injuries 39:1612–1618.

34. Nasereddin A, Shtayeh I, Ramlawi A, Salman N, Salem I, Abdeen Z. 2013. Streptococcus pneumoniae from Palestinian nasopharyngeal carriers: serotype distribution and antimicrobial resistance. PloS one 8.

35. Sjölander I, Hansen F, Elmanama A, Khayyat R, Abu-Zant A, Hussein A, Taha AA, Hammerum AM, Ciofu O. 2014. Detection of NDM-2-producing Acinetobacter baumannii and VIM-producing Pseudomonas aeruginosa in Palestine. Journal of Global Antimicrobial Resistance 2:93–97.

36. Zaid AM. 2013. Distribution of bacterial uropathogens and their susceptibility patterns over twelve years (2001-2013) in Palestine. The International Arabic Journal of Antimicrobial Agents 3.

37. Elmanama AA, Tayyem NEA, Allah SAN. 2014. The bacterial etiology of otitis media and their antibiogram among children in Gaza Strip, Palestine. Egyptian Journal of Ear, Nose, Throat and Allied Sciences 15:87–91.

38. Megged O. 2014. Extended-spectrum β-lactamase-producing bacteria causing community-acquired urinary tract infections in children. Pediatric nephrology (Berlin, Germany) 29:1583–1587.

39. Hijazi N, Elmanama AA, Al-Hindi A. 2009. Vancomycin-resistant enterococci in fecal samples from hospitalized patients and non-hospitalized individuals in Gaza City. Journal of Public Health 17:243–249.

40. Elmanama AA, Al-Aydi IM, Al-Reefi MR. 2020. Biofilm Formation and Methicillin Resistance of Staphylococcus aureus Isolated from Clinical Samples. The International Arabic Journal of Antimicrobial Agents 10.

41. Adwan G, Shaheen H, Adwan K, Barakat A. 2015. Molecular characterization of methicillin resistant Staphylococcus aureus isolated from hospitals environments and patients in Northern Palestine. Epidemiology, Biostatistics and Public Health 12.

42. Abu Taha A, Atia Z, Naji R. 2019. Prevalence and antibiotic susceptibility of bacterial pathogens at a tertiary care hospital in Nablus, occupied Palestinian territory: a cross-sectional survey. The Lancet 393:S50.

43. Laham NA. 2015. Detection and Antibiotic Resistance Pattern of Staphylococcus aureus and MRSA Isolated from Healthcare Workers Nares at Gaza Hospitals, Palestine. The International Arabic Journal of Antimicrobial Agents 5.

44. Hadyeh E, Azmi K, Seir RA, Abdellatief I, Abdeen Z. 2019. Molecular Characterization of Methicillin Resistant Staphylococcus aureus in West Bank-Palestine. Frontiers in public health 7.

45. Rabee HA, Tanbour R, Nazzal Z, Hamshari Y, Habash Y, Anaya A, Iter A, Gharbeyah M, Abugaber D. 2020. Epidemiology of Sepsis Syndrome among Intensive Care Unit Patients at a Tertiary University Hospital in Palestine in 2019. Indian Journal of Critical Care Medicine 24:551.

46. Tayh G, Al Laham N, Ben Yahia H, Ben Sallem R, Elottol AE, Ben Slama K. 2019. Extended-Spectrum β-Lactamases among Enterobacteriaceae Isolated from Urinary Tract Infections in Gaza Strip, Palestine. BioMed Research International 2019.

47. Adwan GM, Owda DM, Abu-Hijleh AA. 2020. Prevalence of Capsular Polysaccharide Genes and Antibiotic Resistance Pattern of Klebsiella pneumoniae in Palestine. Jordan Journal of Biological Sciences 13.

48. Tayh G, Nagarjuna D, Sallem RB, Verma V, Chairat S, Boudabous A, Yadav M, Slama KB. 2020. First report of VIM metallo-β-lactamase production in Escherichia coli and Klebsiella pneumoniae clinical isolates from Gaza Strip, Palestine. Germs 10:18.

49. Laham NA, Mediavilla JR, Chen L, Abdelateef N, Elamreen FA, Ginocchio CC, Pierard D, Becker K, Kreiswirth BN. 2015. MRSA clonal complex 22 strains harboring toxic shock syndrome toxin (TSST-1) are endemic in the primary hospital in Gaza, Palestine. PloS one 10.

50. Segal Z, Cohen MJ, Engelhard D, Tenenbaum A, Simckes AM, Benenson S, Stepensky P, Averbuch D. 2016. Infants under two months of age with urinary tract infections are showing increasing resistance to empirical and oral antibiotics. Acta Paediatrica 105:e156–e160.

51. Tayh G, Laham NA, Elmanama A, SLAMA KB. 2015. Occurrence and antimicrobial susceptibility pattern of ESBL among Gram-negative bacteria isolated from burn unit of Al Shifa hospital in Gaza, Palestine. The International Arabic Journal of Antimicrobial Agents 5:3.

52. Almasri M, Abu Hasan N, Sabbah N. 2016. Macrolide and lincosamide resistance in staphylococcal clinical isolates in Nablus, Palestine. Turkish Journal of Medical Sciences 46:1064–1070.

53. Taha AA, Shtawi A, Jaradat A, Dawabsheh Y. 2018. Prevalence and Risk Factors of Extended Spectrum Beta-Lactamase-Producing Uropathogens among UTI Patients in the Governmental Hospitals of North West Bank: A Cross-Sectional Study. Journal of Infectious Diseases & Preventive Medicine 6.

54. Adwan G, Hasan NA, Sabra I, Sabra D, Al-Butmah S, Odeh S, Albake ZA, Badran H. 2016. Detection of bacterial pathogens in surgical site infections and their antibiotic sensitivity profile. International Journal of Medical Research & Health Sciences 5:75–82.

55. Adwan G, Rabaya D, Adwan K, Al-Sheboul S. 2016. Prevalence of β-lactamases in clinical isolates of Enterobacter cloacae in the West Bank-Palestine. International Journal of Medical Research & Health Sciences 5:49–59.

56. Adwan G, Shtayah A, Adwan K, Al-Sheboul S, Othman S, Purnachandra ), Ganji N. 2016. Prevalence and Molecular Characterization of P. aeruginosa Isolates in the West Bank-Palestine for ESBLs, MBLs and Integrons. Journal of Applied Life Sciences International 8:1–11.

57. El Aila NA, Al Laham NA, Ayesh BM. 2017. Nasal carriage of methicillin resistant Staphylococcus aureus among health care workers at Al Shifa hospital in Gaza Strip. BMC Infectious Diseases 17:1–7.

58. Aila NAE, Esleem SE, Elmanama AA. 2017. Prevalence of Group B Streptococcus Colonization among Pregnant Women in Gaza strip, Palestine. IUG Journal of Natural Studies 25.

59. Abdullah N, Aila E. 2017. Prevalence and molecular characterization of extended-spectrum beta-lactamase producing Escherichia coli and Klebsiella Pneumoniae isolated from Al Shifa hospital, Gaza, Palestine‬. Al-Aqsa University Journal (Natural Sciences Series) 21:44–67.

60. Abdoh Q, Kharraz L, Ayoub K, Khraim J, Awad W, Sbeah A, Turman S. 2018. Helicobacter pylori resistance to antibiotics at the An-Najah National University Hospital: a cross-sectional study. Lancet 391:S32.

61. Elmanama AA. 2018. Bacterial Etiology of Urinary Tract Infection and their Antimicrobial Resistance Profiles. Journal of Al Azhar University-Gaza (Natural Sciences) 20.

62. Rida RH, Al Laham NA, Elmanama AA. 2018. Carbapenem resistance among clinical and environmental Gram-negative isolates recovered from hospitals in Gaza strip, Palestine. Germs 8:147.

63. Taha AA, Daoud A, Zaid S, Sammour S, Belleh M, Daifi R. 2018. Active surveillance for asymptomatic colonisation by multidrug-resistant bacteria in patients transferred to a tertiary care hospital in the occupied Palestinian territory. The Lancet 391:S2.

64. Laham NAA, Elkhair EA, Bashir A, Abdelateef N. 2017. Resistance profiles and biofilm formation of coagulase negative staphylococci isolated from clinical specimens in a tertiary care hospital in Palestine. The International Arabic Journal of Antimicrobial Agents 7.

65. Alkhodari SA, Elmanama AA. 2021. Multidrug Resistance of Uropathogens at Governmental Hospitals in the Gaza Strip/Palestine. The International Arabic Journal of Antimicrobial Agents 11.

66. Qadi M, Abutaha A, Al-Shehab R, Sulaiman S, Hamayel A, Hussein A, Abutaha S, Dawoud A, Hussein F. 2021. Prevalence and Risk Factors of Group B Streptococcus Colonization in Pregnant Women: A Pilot Study in Palestine. Canadian Journal of Infectious Diseases and Medical Microbiology 2021.

67. Elmanama AA, El-Aydi I, Al-Reefi M, Ferwana N. 2022. Antibiogram of bacterial isolates from clinical specimens during 2018-2020 at Al-Aqsa hospital, Gaza, Palestine. The International Arabic Journal of Antimicrobial Agents 12.

68. Galper E, Bdolah-Abram T, Megged O. 2021. Assessment of infections rate due to community-acquired Methicillin-resistant Staphylococcus aureus and evaluation of risk factors in the paediatric population. Acta paediatrica (Oslo, Norway : 1992) 110:1579–1584.

69. Qamar AKA, Habboub TM, Elmanama AA. 2022. Antimicrobial resistance of bacteria isolated at the European Gaza Hospital before and after the Great March of Return protests: a retrospective study. The Lancet 399:S14.

70. Qadi M, Alhato S, Khayyat R, Elmanama AA. 2021. Colistin Resistance among Enterobacteriaceae Isolated from Clinical Samples in Gaza Strip. Canadian Journal of Infectious Diseases and Medical Microbiology 2021.

71. Qadi M, Khayyat R, AlHajhamad MA, Naji YI, Maraqa B, Abuzaitoun K, Mousa A, Daqqa M. 2021. Microbes on the Mobile Phones of Healthcare Workers in Palestine: Identification, Characterization, and Comparison. Canadian Journal of Infectious Diseases and Medical Microbiology 2021.

72. Tayh G, Al Laham N, Fhoula I, Abedelateef N, El-Laham M, Elkader Elottol A, Ben Slama K. 2021. Frequency and Antibiotics Resistance of Extended-Spectrum Beta-Lactamase (ESBLs) Producing Escherichia coli and Klebsiella pneumoniae Isolated from Patients in Gaza Strip, Palestine. Journal of Medical Microbiology and Infectious Diseases 9:133–141.

73. Aish KAE, Fayad H, Arkan RE, Farwana N, Saquer N. 2022. Antimicrobial resistance in bacteria isolated from blood cultures from babies with sepsis in neonatal intensive care units in the Gaza Strip: a cross-sectional study. The Lancet 399:S12.

74. Al-Afifi A, Aish KAE, Jbour HE, Helless M. 2022. Identifying the characteristics of antibacterial resistance in urine specimens of children in the Gaza Strip: a hospital-based cross-sectional study. The Lancet 399:S11.

75. Awwad E, Srour M, Hasan S, Khatib S. 2022. Molecular determination, serotyping, antibiotic profile and virulence factors of group B Streptococcus isolated from invasive patients at Arabcare Hospital Laboratory, Palestine. American journal of infection control 50:934–940.

76. El Aila NA, Al Laham NA, Ayesh BM. 2023. Prevalence of extended spectrum beta lactamase and molecular detection of blaTEM, blaSHV and blaCTX-M genotypes among Gram negative bacilli isolates from pediatric patient population in Gaza strip. BMC infectious diseases 23:99.

77. Al Laham NA. 2012. Distribution and Antimicrobial Resistance Pattern of Bacteria Isolated from Operation Theaters at Gaza Strip. Journal of Al Azhar University-Gaza (Natural Sciences) 14:19–34.

78. Abu Al-Halawa D, Sarama R, Abdeen Z, Qasrawi R. 2019. Knowledge, attitudes, and practices relating to antibiotic resistance among pharmacists: a cross-sectional study in the West Bank, Palestine. The Lancet 393:S7.

79. Sawalha A. 2010. Extent of storage and wastage of antibacterial agents in Palestinian households. Pharmacy World and Science 32:530–535.

80. Sawalha AF. 2008. Self-medication with antibiotics: A study in Palestine. International Journal of Risk & Safety in Medicine 20:213–222.

81. Ayoub S, Musalam A, Mahadi AAA. 2017. Drug utilization in primary healthcare centres in the Gaza Strip. Eastern Mediterranean Health Journal 23:649–656.

82. Musmar SM, Ba’Ba H, Owais A. 2014. Adherence to guidelines of antibiotic prophylactic use in surgery: A prospective cohort study in North West Bank, Palestine. BMC Surgery 14:1–7.

83. Alfaqawi M, Abuowda Y, Böttcher B, Alserr K, Elmassry AE. 2021. Antibiotic use in acute upper respiratory tract infections and uncomplicated lacerations in the Gaza Strip: clinical audit and re-audit. The Lancet 398:S7.

84. Pigeolet M, Hababeh M, Khader A, Seita A, Coenen S. 2018. The effect of continuity of care on antibiotics prescription for Palestinian refugees in UNRWA health centres: a cross-sectional study. The Lancet 391:S19.

85. Zyoud SH, Shtaya RM, Hamadneh DQ, Sawalmeh SN, Khadrah HA, Zedat RR, Othman A, Sweileh WM, Awang R, Al-Jabi SW. 2020. Parental knowledge, attitudes, and practices towards self-medication for their children: A cross-sectional study from Palestine. Asia Pacific Family Medicine 18.

86. Abu Taha A, Abu-Zaydeh AH, Ardah RA, Al-Jabi SW, Sweileh WM, Awang R, Zyoud SH. 2016. Public Knowledge and Attitudes Regarding the Use of Antibiotics and Resistance: Findings from a Cross-Sectional Study Among Palestinian Adults. Zoonoses and Public Health 63:449–457.

87. Zyoud SH, Taha AA, Araj KF, Abahri IA, Sawalha AF, Sweileh WM, Awang R, Al-Jabi SW. 2015. Parental knowledge, attitudes and practices regarding antibiotic use for acute upper respiratory tract infections in children: A cross-sectional study in Palestine. BMC Pediatrics 15:1–9.

88. McGirk J. 2011. Norway investigates Gaza’s drug crisis. The Lancet 377:1225–1226.

89. Elmanama AA, Al-Refi MR, Jaber AH, Sukkar IM. 2019. Vancomycin-Resistant Enterococcus spp. (VRE) from Chicken cloacal Swab in Gaza Strip Poultry Farms. IUG Journal of Natural Studies 27.

90. Elmanama AA, Al-Reefi MR, Shamali MA, Hemaid HI. 2019. Carbapenem-resistant Gram-negative bacteria isolated from poultry samples: a cross-sectional study. The Lancet 393:S21.

91. Qabajah MH. 2011. Avian Pathogenic Escherichia coli (APEC) in Palestine: Characterization of Virulence Factors and Antibiotic Resistance Profile. Bethlehem University, Bethlehem.

92. Adwan GM, Adwan KM. 2004. Isolation of shiga toxigenic Escherichia coli from raw beef in Palestine. International Journal of Food Microbiology 97:81–84.

93. Adwan G, Isayed H. 2018. Prevalence and Characterization of Staphylococcus aureus Isolated from Bulk Tank Milk Dairy Cow Farms in West Bank-Palestine Virulence Genes in Escherichia coli View project Plant genotoxixity View project. Article in Microbiology Research Journal International https://doi.org/10.9734/MRJI/2018/40074.

94. Adwan GM. 2006. Antibiotic resistance against Staphylococcal isolates recovered from subclinical mastitis in the north of Palestine. IUG Journal for Natural Studies 14.
