# Supplementary material for: Antimicrobial resistance in a protracted war setting: a review of the literature from Palestine
Source: mSystems. 2025 May 21;10(6):e01679-24. doi: 10.1128/msystems.01679-24 (PMC12172458; doi:10.1128/msystems.01679-24)
Supplement: Supplemental material — Sample search strategy. [file msystems.01679-24-s0002.docx]

Final Searches

Antimicrobial resistance in Palestine Scoping Review

**PubMed: 193**

(Palestin*[tw] OR Gaza[tw] OR "West Bank"[tw] OR Jerusalem[tw] OR "conflict affected"[tw]) AND ("Drug Resistance, Microbial"[mesh] OR "Antimicrobial Stewardship"[mesh] OR "Drug Resistance, Multiple"[mesh] OR (("Anti-Bacterial Agents"[mesh] OR "beta-Lactams"[mesh] OR Methicillin[mesh] OR "Fluoroquinolones"[mesh] OR Cephalosporins[mesh] OR "beta-Lactamases"[mesh] OR Vancomycin[mesh]) AND "Drug Resistance"[mesh:noexp]) OR "Drug Collateral Sensitivity"[mesh] OR "Methicillin-Resistant Staphylococcus aureus"[mesh] OR "Methicillin Resistance"[mesh] OR "Carbapenem-Resistant Enterobacteriaceae"[mesh] OR "Cephalosporin Resistance"[mesh] OR "beta-Lactam Resistance"[mesh] OR "Vancomycin Resistance"[mesh] OR "Vancomycin-Resistant Enterococci"[mesh] OR "Vancomycin-Resistant Staphylococcus aureus"[mesh] OR ((antibiotic*[tw] OR antimicrob*[tw] OR anti-microb*[tw] OR antibacter*[tw] OR anti-bacter*[tw] OR vancomycin[tw] OR fluoroquinolone*[tw] OR cephalosporin*[tw] OR MRSA[tw] OR methicillin[tw] OR carbapenem*[tw] OR metallo-beta-lactamase[tw] OR ESBL[tw] OR beta-lactamase*[tw] OR multi-drug[tw] OR multidrug[tw] OR bacteri*[tw] OR microb*[tw]) AND (resist*[tw] OR sensitiv*[tw] OR suscept*[tw] OR efficac*[tw] OR non-suscept*[tw] OR nonsuscept*[tw])))

**Cochrane: 7**

ID Search Hits

#1 (Palestin* OR Gaza OR "West Bank" OR Jerusalem OR "conflict affected"):ti,ab,kw 402

#2 MeSH descriptor: [Drug Resistance, Microbial] explode all trees 2381

#3 MeSH descriptor: [Antimicrobial Stewardship] explode all trees 53

#4 MeSH descriptor: [Drug Resistance, Multiple] explode all trees 329

#5 MeSH descriptor: [Drug Collateral Sensitivity] explode all trees 0

#6 MeSH descriptor: [Methicillin-Resistant Staphylococcus aureus] explode all trees 236

#7 MeSH descriptor: [Methicillin Resistance] explode all trees 118

#8 MeSH descriptor: [Carbapenem-Resistant Enterobacteriaceae] explode all trees 5

#9 MeSH descriptor: [Cephalosporin Resistance] explode all trees 4

#10 MeSH descriptor: [beta-Lactam Resistance] explode all trees 368

#11 MeSH descriptor: [Vancomycin Resistance] explode all trees 24

#12 MeSH descriptor: [Vancomycin-Resistant Enterococci] explode all trees 11

#13 MeSH descriptor: [Vancomycin-Resistant Staphylococcus aureus] explode all trees 0

#14 #2 OR #3 OR #4 OR #5 OR #6 OR #7 OR #8 OR #9 OR #10 OR #11 OR #12 OR #13 2775

#15 MeSH descriptor: [Anti-Bacterial Agents] explode all trees 13078

#16 MeSH descriptor: [beta-Lactams] explode all trees 9873

#17 MeSH descriptor: [Methicillin] explode all trees 102

#18 MeSH descriptor: [Fluoroquinolones] explode all trees 3845

#19 MeSH descriptor: [Cephalosporins] explode all trees 4498

#20 MeSH descriptor: [beta-Lactamases] explode all trees 93

#21 MeSH descriptor: [Vancomycin] explode all trees 866

#22 MeSH descriptor: [Drug Resistance] explode all trees 10137

#23 #15 OR #16 OR #17 OR #18 OR #19 OR #20 OR #21 21751

#24 #23 AND #22 1291

#25 (antibiotic* OR antimicrob* OR anti-microb* OR antibacter* OR anti-bacter* OR vancomycin OR fluoroquinolone* OR cephalosporin* OR MRSA OR methicillin OR carbapenem* OR metallo-beta-lactamase OR ESBL OR beta-lactamase* OR multi-drug OR multidrug OR bacteri* OR microb*):ti,ab,kw 85304

#26 (resist* OR sensitiv* OR suscept* OR efficac* OR non-suscept* OR nonsuscept*):ti,ab,kw 531023

#27 #25 AND #26 35793

#28 #24 OR #27 OR #14 36491

#29 #28 AND #1 7

**CINAHL+ Complete (via Harvard): 1,384**

#1: TX (Palestin* OR Gaza OR "West Bank" OR Jerusalem OR "conflict affected")

#2: ((MH "Drug Resistance, Microbial+") OR (MH "Antimicrobial Stewardship") OR (((MH "Drug Resistance+") OR (MH "Antibiotics+")) AND (MH "Antiinfective Agents+")) OR (MH "Methicillin-Resistant Staphylococcus Aureus") OR (MH "Vancomycin-Resistant Staphylococcus Aureus") OR (MH "Carbapenem-Resistant Enterobacteriaceae") )

#3: TX ( antibiotic* OR antimicrob* OR anti-microb* OR antibacter* OR anti-bacter* OR vancomycin OR fluoroquinolone* OR cephalosporin* OR MRSA OR methicillin OR carbapenem* OR metallo-beta-lactamase OR ESBL OR beta-lactamase* OR multi-drug OR multidrug OR bacteri* OR microb* ) AND TX ( resist* OR sensitiv* OR suscept* OR efficac* OR non-suscept* OR nonsuscept* )

#4: #2 OR #3

#5: #4 AND #1

**Embase: 361**

**#1:** 'palestine'/exp OR 'palestine' OR 'gaza strip palestine'/exp OR 'gaza strip palestine' OR 'gaza strip'/exp OR 'gaza strip' OR palestin*:ab,ti,kw OR gaza:ab,ti,kw OR 'west bank':ab,ti,kw OR jerusalem:ab,ti,kw OR 'conflict affected':ab,ti,kw

**#2:** 'antibiotic sensitivity'/exp OR ('antiinfective agent'/exp AND 'drug resistance'/exp) OR 'antimicrobial stewardship'/exp OR 'collateral sensitivity'/exp OR 'methicillin resistant staphylococcus aureus'/exp OR 'carbapenem-resistant enterobacteriaceae'/exp OR 'vancomycin resistant enterococcus'/exp OR 'vancomycin resistant staphylococcus aureus'/exp OR ((antibiotic*:ab,ti,kw OR antimicrob*:ab,ti,kw OR 'anti microb*':ab,ti,kw OR antibacter*:ab,ti,kw OR 'anti bacter*':ab,ti,kw OR vancomycin:ab,ti,kw OR fluoroquinolone*:ab,ti,kw OR cephalosporin*:ab,ti,kw OR mrsa:ab,ti,kw OR methicillin:ab,ti,kw OR carbapenem*:ab,ti,kw OR 'metallo beta lactamase':ab,ti,kw OR esbl:ab,ti,kw OR 'beta lactamase*':ab,ti,kw OR 'multi drug':ab,ti,kw OR multidrug:ab,ti,kw OR bacteri*:ab,ti,kw OR microb*:ab,ti,kw) AND (resist*:ab,ti,kw OR sensitiv*:ab,ti,kw OR suscept*:ab,ti,kw OR efficac*:ab,ti,kw OR 'non suscept*':ab,ti,kw OR nonsuscept*:ab,ti,kw))

**#3**: #1 AND #2

**Web of Science Core Collection: 157**

#1. TS=(Palestin* OR Gaza OR "West Bank" OR Jerusalem OR "Conflict affected")

#2. TI=(Palestin* OR Gaza OR "West Bank" OR Jerusalem OR "Conflict affected")

#3. AB=(Palestin* OR Gaza OR "West Bank" OR Jerusalem OR "Conflict affected")

#4. #1 OR #2 OR #3

#5. TS=((antibiotic* OR antimicrob* OR anti-microb* OR antibacter* OR anti-bacter* OR vancomycin OR fluoroquinolone* OR cephalosporin* OR MRSA OR methicillin OR carbapenem* OR metallo-beta-lactamase OR ESBL OR beta-lactamase* OR multi-drug OR multidrug OR bacteri* OR microb*) AND (resist* OR sensitiv* OR suscept* OR efficac* OR non-suscept* OR nonsuscept*))

#6: TI=((antibiotic* OR antimicrob* OR anti-microb* OR antibacter* OR anti-bacter* OR vancomycin OR fluoroquinolone* OR cephalosporin* OR MRSA OR methicillin OR carbapenem* OR metallo-beta-lactamase OR ESBL OR beta-lactamase* OR multi-drug OR multidrug OR bacteri* OR microb*) AND (resist* OR sensitiv* OR suscept* OR efficac* OR non-suscept* OR nonsuscept*))

#7. AB=((antibiotic* OR antimicrob* OR anti-microb* OR antibacter* OR anti-bacter* OR vancomycin OR fluoroquinolone* OR cephalosporin* OR MRSA OR methicillin OR carbapenem* OR metallo-beta-lactamase OR ESBL OR beta-lactamase* OR multi-drug OR multidrug OR bacteri* OR microb*) AND (resist* OR sensitiv* OR suscept* OR efficac* OR non-suscept* OR nonsuscept*))

**Google Scholar:** first 100 results from each search below **= 400**

(Palestine OR "Gaza Strip" OR "West Bank" OR Jerusalem) AND (antibiotic OR antibacterial OR antimicrobial OR microbial OR bacterial) AND (resistance OR resistant)

(Palestine OR "Gaza Strip" OR "West Bank" OR Jerusalem) AND (antibiotic OR antibacterial OR antimicrobial OR microbial OR bacterial) AND (Sensitivity OR susceptibility)

(Palestine OR "Gaza Strip" OR "West Bank" OR Jerusalem) AND (vancomycin OR methicillin OR Cephalosporin OR fluoroquinolone OR carbapenem OR beta-lactamase) AND (Resistance OR resistant)

(Palestine OR "Gaza Strip" OR "West Bank" OR Jerusalem) AND (vancomycin OR methicillin OR Cephalosporin OR fluoroquinolone OR carbapenem OR beta-lactamase) AND (susceptibility OR sensitivity)

Total before deduplcation: 2,502

Duplicates found in EndNote: 715

Duplicates found in Covidence: 2

Total for review: 1,785
